# Functional impact of multi-omic interactions in lung cancer

**DOI:** 10.3389/fgene.2024.1282241

**Published:** 2024-02-08

**Authors:** Miguel Ángel Díaz-Campos, Jorge Vasquez-Arriaga, Soledad Ochoa, Enrique Hernández-Lemus

**Affiliations:** ^1^ Computational Genomics Division, National Institute of Genomic Medicine, Mexico City, Mexico; ^2^ Department of Obstetrics and Gynecology, Cedars-Sinai Medical Center, Los Angeles, CA, United States; ^3^ Center for Complexity Sciences, Universidad Nacional Autónoma de México, Mexico City, Mexico

**Keywords:** lung adenocarcinoma, lung squamous cell carcinoma, multiomics, mutual information, network construction, computational analysis

## Abstract

Lung tumors are a leading cause of cancer-related death worldwide. Lung cancers are highly heterogeneous on their phenotypes, both at the cellular and molecular levels. Efforts to better understand the biological origins and outcomes of lung cancer in terms of this enormous variability often require of high-throughput experimental techniques paired with advanced data analytics. Anticipated advancements in multi-omic methodologies hold potential to reveal a broader molecular perspective of these tumors. This study introduces a theoretical and computational framework for generating network models depicting regulatory constraints on biological functions in a semi-automated way. The approach successfully identifies enriched functions in analyzed omics data, focusing on Adenocarcinoma (LUAD) and Squamous cell carcinoma (LUSC, a type of NSCLC) in the lung. Valuable information about novel regulatory characteristics, supported by robust biological reasoning, is illustrated, for instance by considering the role of genes, miRNAs and CpG sites associated with NSCLC, both novel and previously reported. Utilizing multi-omic regulatory networks, we constructed robust models elucidating omics data interconnectedness, enabling systematic generation of mechanistic hypotheses. These findings offer insights into complex regulatory mechanisms underlying these cancer types, paving the way for further exploring their molecular complexity.

## 1 Introduction

Lung cancer (LC) is one of the most prevalent and deadliest forms of cancer globally–ranks second for cancer incidence and first for cancer mortality–. It is responsible for the highest cancer mortality rates worldwide. LC is broadly categorized into two primary types: non-small cell lung cancer (NSCLC) and small cell lung cancer (SCLC), with NSCLC being the more prevalent form (Huang et al., 2022). NSCLC accounts for approximately 85% of all lung cancer cases and exhibits a particularly low 5-year survival rate, estimated at just 13%. The remaining 15% of cases are attributed to SCLC. Moreover, within the NSCLC category, three primary histopathological subtypes are recognized. These subtypes include adenocarcinoma (LUAD), comprising 45%–50% of NSCLC cases, squamous cell carcinoma (LUSC), with a prevalence of 30%–35%, and large cell (undifferentiated) carcinoma, accounting for 5%–10% of cases (Wang et al., 2022). LC often manifests with subtle early symptoms, and it is frequently diagnosed at an advanced stage, rendering treatment more complex. Typical indicators may include persistent coughing, chest discomfort, breathing difficulties, unexplained weight loss, fatigue, and recurrent respiratory infections (Dritsas and Trigka, 2022). The treatment approaches for lung cancer hinge on several factors, including the cancer type, stage, and the overall health status of the patient. These therapeutic modalities may involve surgical intervention, radiation therapy, chemotherapy, targeted therapies, and immunotherapy. Timely detection and the application of effective treatment are decisive in enhancing survival rates and the overall quality of life for individuals grappling with lung cancer (Araghi et al., 2023).

Numerous diagnostic approaches have been proposed for identifying lung cancer subtypes, encompassing methods like computed tomography (CT) and pathological examination. In recent years, as sequencing technologies have advanced, liquid biopsy has emerged as a non-invasive and efficient means of early cancer detection and targeted therapy (Crowley et al., 2013; Howlader et al., 2020). Furthermore, diverse techniques operating at various biological levels have been used, such as the assessment of single nucleotide variations, DNA methylation, and quantifying miRNA expression (Hao et al., 2017; Ahmed et al., 2022; Wang et al., 2022).

In the context of contemporary biomedical research, high-throughput technologies have sparked a revolution by facilitating large-scale genome-wide association studies and enabling the exploration of global transcript levels. Additionally, the integration of multi-omics data in cancer research has provided a systems biology approach, leveraging the synergies between diverse molecular descriptions. Nevertheless, the pursuit of comprehensive mechanistic insights remains an ongoing challenge (Hasin et al., 2017; Argelaguet et al., 2018). To construct comprehensive genomic and transcriptomic regulatory maps able to capture lung cancer complexity, we need to analyze numerous gene expressions and high-dimensional genetic variants. This process typically involves several approaches to multivariate regression analysis (Harpole Jr et al., 1995; Farhangfar et al., 2014), interestingly, genetic regulatory connections are inherently sparse, with a single variant influencing only a small fraction of gene expressions (Zhou et al., 2016).

In the field of bioinformatics research, numerous multiomics methods have been introduced, to name a few, J. Wang et al. (Wang et al., 2022) developed a precise multiomics risk model for predicting Tumor Mutational Burden (TMB) in patients with LUAD. This model integrated gene/miRNA expression and DNA methylation data sourced from The Cancer Genome Atlas (TCGA). By considering these multiomic features, the model was able to capture subtle alterations within the tumor microenvironment, leading to a more accurate prediction of TMB. On a related note (Song et al., 2023), conducted a comprehensive investigation into the impact of Intratumor Heterogeneity (ITH) on the effectiveness of bispecific antibody (bsAb) immunotherapy in patients with advanced NSCLC. Their study leveraged advanced techniques, such as Digital Spatial Profiling (DSP), Next-Generation Sequencing (NGS), and the nCounter platform, to analyze transcriptomic and proteomic data derived from over 100 Regions of Interest (ROIs). Multiomic approaches have been succesfully applied to improve prognostics on a number of neoplasms, such as colon (Yang et al., 2020), liver (Chaudhary et al., 2018) and breast cancer (Xie et al., 2018), even PanCancer studies have been developed (Chai et al., 2021).

In view of these facts, here we resort to Sparse Generalized Canonical Correlation Analysis (SGCCA) to analyze DNA methylation, gene expression, and miRNA from LUAD and LUSC data from TCGA. SGCCA, a potent statistical method with LASSO penalization (Tenenhaus et al., 2014), which identifies correlated features in extensive datasets. SGCCA was coupled with ARACNE (Margolin et al., 2006) to reveal features and their interconnections and evaluate the role of relevant methylation sites, miRNAs and mRNAS in oncogenic mechanisms. In summary, in this article, we probe these integrative approaches to explain intricate biological complexities in the context of lung cancer.

## 2 Methods

**FIGURE 1:**
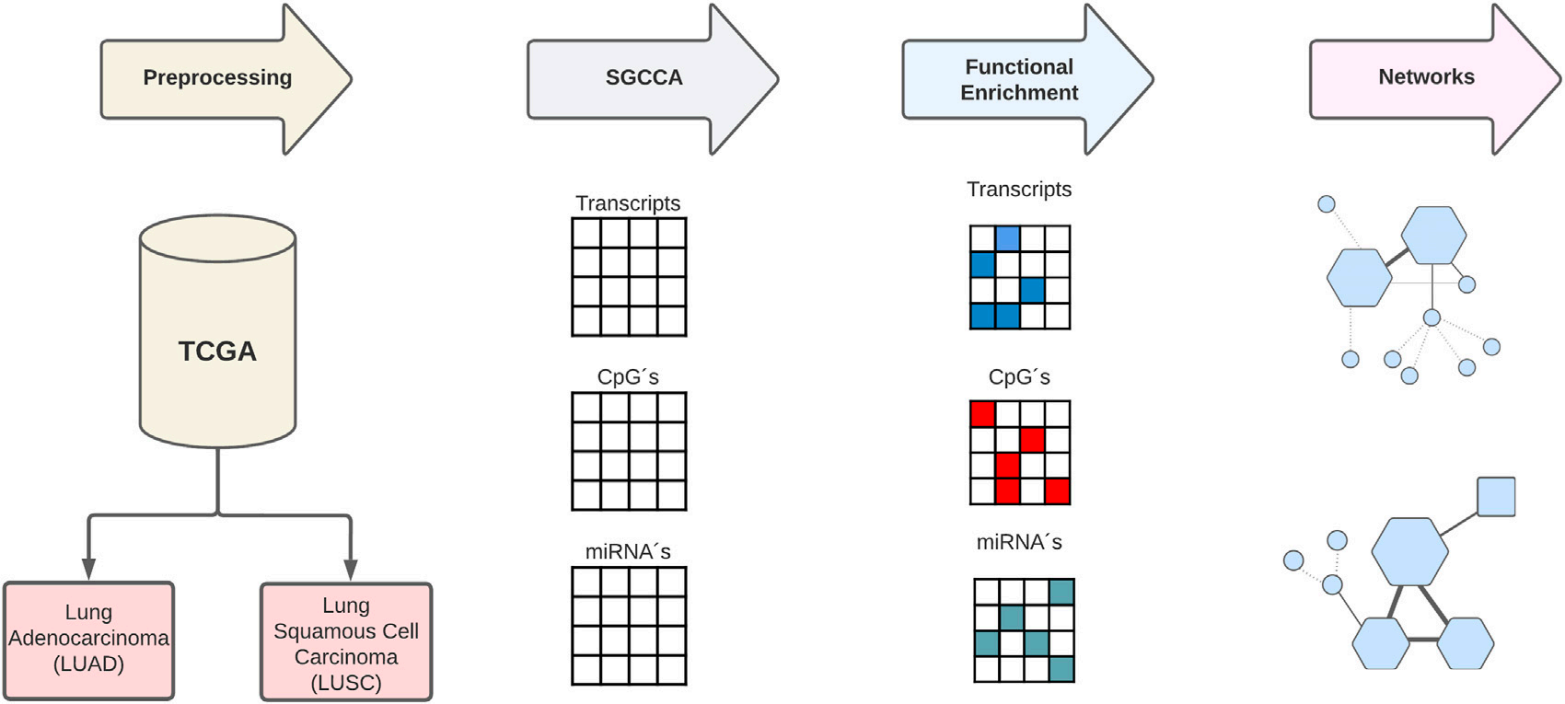
Summary of the procedure.

The following analyses (see Figure 1) were performed with R programming language version 4.3.0 (R Core Team, 2022) and can be found on the GitHub repository at https://github.com/arriagajorge/Lung-76-cancer-multiomics.

### 2.1 Acquisition and preprocessing data

The TCGAbiolinks package (Silva et al., 2017) was employed to acquire the TCGA dataset. For our study, we selected samples from unique patients with concurrent Illumina Human Methylation 450, RNA-seq, and miRNA-seq data. These criteria limited our sample size to 188 for the Lung Adenocarcinoma subtype (LUAD) with 5 samples from normal tissue and 72 for the Lung Squamous Cell Carcinoma subtype (LUSC) with 3 samples from normal tissue.

Samples for the methylation analysis were obtained with the Infinium HumanMethylation450 BeadChip, which covers 99% of RefSeq genes at transcription repressive sites around promoters and transcription favorable sites (Zhou et al., 2017; Wang et al., 2018). Since we measured three distinct techniques, methylation beadchip, RNAseq, and miRNAseq, we treated them as separate omics entities referred to as CPG sites, transcripts, and miRNAs. Incorporating the entire set of features, our aim was to capture the highest number of interactions possible. Subtype classification per cancer type was also downloaded from the GDC metadatada (Colaprico et al., 2016; Mounir et al., 2019) Consequently, GDC samples were manually merged, to achieve a satisfactory sample size into LUAD and LUSC subtypes.

Omics preprocessing was performed according to published guidelines (Dedeurwaerder et al., 2011) using biomaRT. The initial step involved the normalization of the transcripts for length and GC content by using the full method, content biases with the NOIseq package (Tarazona et al., 2015) and EDASeq package (Risso et al., 2011) full normalization.

Transcripts and miRNAs with zero counts at the low counts filter were removed, normalization between samples was performed using TMM (Trimmed Mean of M-values) (Robinson and Oshlack, 2010). Batch effect was corrected from samples using ARSyNseq by removing not associated systematic noise with the cancer type samples (Nueda et al., 2012). microRNAs preprocessing follows the same steps, excluding any considerations for length or GC bias, and making use of the median method for sample normalization (Tam et al., 2015).

For the CpG analysis, probes that exhibited more than 25% missing values were eliminated. Transcripts coding for transcription factors (TF-genes) were tagged using downloaded annotation data. Finally, nearest neighbor imputation was applied to fill in the remaining missing values, transforming the data into M-value matrices.

### 2.2 Sparse Generalized Canonical Correlation Analysis (SGCCA)

We applied normalization to each omic by dividing by the square root of the first eigenvalue, after completing the preprocessing phase. Subsequently, we merged the normalized omics data on a patient-by-patient fashion, creating one matrix per lung cancer type. This way, we guarantee that the impact of each omic on the successive analysis is determined by its relative variance (Voillet et al., 2016).

The SGCCA method was conducted by using the mixOmics package (Rohart et al., 2017). This method was followed exclusively in cancer samples, i.e., excluding normal samples. The analysis was performed by providing the algorithm with the different blocks of data, a corresponding sparsity parameter, along with the number of components to recover (ncomp), a design matrix, and a covariance-maximizing function. Cross validation with *k* = 5 was employed to select sparsity parameters for each omic taking the sequence [0.01, 0.02, …, 0.99] (Ochoa and Hernández-Lemus, 2023).

In each iteration, SGCCA was executed, retrieving a single component, recording the chosen number of features and the average variance explained (AVE) (Supplementary Figure S1). Sparsity parameters were carefully selected to achieve the highest AVE with the fewest number of features per cancer type, taking 0.01 for CpG sites, 0.02 for transcripts and 0.11 for microRNAS in LUSC type, while taking 0.30 for CpG sites, 0.01 for transcripts and 0.14 for microRNAS in LUAD type. To equilibrate the variation among the different values, eigenvalue normalization was performed, ensuring a balanced representation. Additionally, separate penalization methods were applied, considering the varying signal sizes observed in the distinct omics.

Finally, SGCCA analysis was executed for each cancer type by using the fitted values and notice that as the sparsity value decreases, the number of selected features also decreases. In the case of each cancer type, the value of ncomp was set to the number of samples minus 1, and the default design matrix was utilized with the centroid function, enabling the incorporation of negative correlation. Due to the application of LASSO penalization, the feature selection by SGCCA is susceptible to some degree of instability. To replicate the filtering method in miRDriver (Bose and Bozdag, 2019), we conducted 100 iterations of SGCCA for each lung cancer type, employing random subsets, consisting of half of the samples each time. Subsequently we retained only those features that were selected in at least 70% of iterations (Supplementary Figure S2).

### 2.3 Functional enrichment analysis

The SGCCA results produced a sparse matrix consisting of loadings that represent the contribution of each feature to every component, with non-zero loadings, which can be examined for functional enrichment. All the features were mapped, including CpG probes, miRNA precursors, and transcripts, to Entrez gene IDs, with direct annotation available for transcripts and miRNAs at Entrez, and for translating CpG probes to Entrez IDs, we obtained the genes affected by each probe from the microarray annotation file. The group of features with non-zero loadings in all SGCCA components was analyzed separately using an over-representation analysis, with Entrez IDs used as input.

Enrichment analyses were performed using the clusterProfiler package (Yu et al., 2012) in the KEGG database (Kanehisa et al., 2017) and biological processes in the gene ontology (Consortium, 2021). Over-representation testing was conducted on functions that exhibited exclusive enrichment in a single dataset. To achieve this, we grouped the exclusively enriched functions based on GO slim and KEGG classes.

The resulting *p*-values from the enrichment were adjusted for multiple testing using the Bonferroni method, then, the association between the grouped categories and the subtypes was evaluated using Fisher’s test. We executed a separate gene set enrichment analysis (GSEA) (Shi and Walker, 2007) using only transcript data to investigate functions affected by differential expression, while using the clusterProfiler package to execute the GSEA without applying a *p*-value cutoff, to obtain GSEA enrichment scores for each of the functions that were over-represented in the SGCCA results.

Finally, the obtained scores revealed whether functions were over-represented among the features associated with different omics and enriched among genes that exhibited altered expression (Supplementary Tables S1–4).

### 2.4 Network construction and analysis

Then, the selected functions were represented as a network to construct potential regulatory models. As a result, we obtained all the features that covaried with the features responsible for the functional enrichment, to target this set (Supplementary Figure S3).

We estimate the mutual information (MI) between each pair of nodes by running ARACNE-multicore (https://github.com/josemaz/aracne-multicore), a recent version of the algorithm developed by Margolin et al. (2006). ARACNE-multicore works in parallel to accelerate the estimation of mutual information between gene pairs. This allowed us to remove all pairs with MI lower than the median value for known regulatory interactions.

Then, we extracted a submatrix from the original dataset and ran ARACNE-multicore to identify regulatory interactions. To capture CpG interactions, we retrieved information from the microarray annotation file, considering the potential influence of position overlap on gene expression. For transcripts, we utilized TFtargets package https://github.com/slowkow/tftargets and for miRNAs, we employed the multiMiR package (Ru et al., 2014).

The infotheo package (Meyer, 2008) was used to calculate mutual information (MI) values for the regulatory interactions. Instead of estimating all pairs involving a specific feature in the matrix (as ARACNE does), we decided to focus on a specific set of predetermined pairs. The threshold was determined for the regulatory interactions by selecting the median MI values instead of the mean to prevent outliers from dominating from having a superior influence on the threshold determination. Considering MI values to vary across different types of pairs, we obtained different thresholds for CpG-transcript, CpG-miRNA, TF transcript-transcript, and miRNA-transcript edges in similar way as in (Ochoa and Hernández-Lemus, 2023).

Comparation of MI values distribution between different types of edges was developed using Kolmogorov-Smirnov test, we decide to choose the lowest median MI from the regulatory interactions when the distribution showed no significant differences, as the single threshold. This allowed more MI interactions to be included in the final network.

For the MI network visualization, we employed cytoscape (Otasek et al., 2019) by using RCy3 package (Gustavsen et al., 2019) and for analysis we used the igraph package (Csardi and Nepusz, 2006). For miRNA differential expression analysis we performed eBayes limma functions (Smyth, 2004) and normalized using voom (Law et al., 2014). Due to the absence of previous batch-effect correction in the methylation data, we employed the missMethyl package (Phipson et al., 2015) in differential analysis to mitigate systematic errors (Marabita et al., 2013).

Making use of Pubmed databases we searched for biological roles associated with each neighbor of a functional node, interactions between node pairs and the databases containing predicted regulatory links using multiMir package. This approach constructed a regulatory model for the functions enriched in the SGCCA through a satisfactorily automated manner.

### 2.5 Analysis of central and topological measures within the gene expression networks

With the information obtained through the networks, we activated the NetworkAnalyzer tool. This tool served as a resource for quantifying key metrics pertaining to network topology. Our ensuing analysis focused on the Average Shortest Path Length, Betweenness Centrality, Closeness Centrality, Neighborhood Connectivity, and Topological Coefficients. The Average Shortest Path Length metric computed the average of the shortest paths between all pairs of nodes, offering insight into the overall efficiency of information transfer within the network. Activating the Betweenness Centrality metric allowed us to identify nodes crucial for information flow, acting as essential links in the network. Enabling Closeness Centrality aided us in identifying nodes with shorter average distances to others, signifying their centrality in the network. The Neighborhood Connectivity parameter explore into the local connectivity of nodes within immediate neighborhoods, uncovering nodes with pronounced local influence. Topological Coefficients provided insights into the influence of nodes on the overall network structure, with higher coefficients indicating a more significant role in maintaining network integrity. We interpreted these metrics in conjunction with biological reports for each transcript and miRNA reported to identify key nodes and their potential implications in the molecular mechanisms for LUAD and LUSC.

### 2.6 CpG sites identification and measurement

For the identification of CpG sites as promoters or enhancers we loaded the genomic annotation information using the TxDb.Hsapiens.UCSC.hg38.knownGene package (Huang et al., 2022) and extracted promoter regions located 1,000 base pairs upstream and up to the transcription start site. Additionally, we obtained enhancer coordinates from the ENCODE project (de Souza, 2012) stored in a BED file (“ENCFF596CUU.bed.gz”) using the data.table package. Subsequently, we converted the enhancer and promoter data into GRanges formats for efficient intersection analysis. To enhance the compatibility of the datasets, we created data frames from the GRanges objects, specifying element types as “promoter” or “enhancer” and combining the information into a unified dataframe. The resulting dataframe included essential details such as genomic coordinates, width, strand, and element type. For further analysis, we focused on CpG sites associated with genes of interest. Leveraging the Ensembl database through the biomaRt package, we obtained the Ensembl transcript IDs (ENST IDs) for the list of selected CpG sites related to LUAD and LUSC data. The retrieved ENST IDs were then merged with the previously generated dataframe containing promoter and enhancer information. The final output is a consolidated dataset that associates CpG sites with their respective promoters and enhancers information for further downstream analysis and interpretation.

To calculate the distances between key CpG sites and the main node genes, we first converted the genomic coordinates of both genes and CpG sites into numeric values. We implemented a midpoint function, facilitating the determination of the midpoint for a given genomic region, which was crucial for distance calculations. Applying this function, we calculated the midpoint of the main genes for each network. To ensure consistency, gene names were uniformly converted to lowercase. Distances were then systematically calculated between the midpoint of the central gene and the midpoints of CpG sites associated with each LUAD and LUSC network. The results were organized into a distance data frame, where each row represented a target CpG site and its corresponding distance from the main gene node.

### 2.7 Cox regression analysis

To explore potential clinical implications, we employed a Cox regression analysis utilizing patient survival data to evaluate the significance of gene interactions specifically associated with the onset of LUSC or LUAD. Survival data from individuals diagnosed with LUSC and LUAD were assembled, and gene interactions linked were systematically identified. These identified interactions were subsequently integrated as explanatory variables within the Cox regression model. Our hypothesis asserts that these gene interactions would demonstrate statistical significance in the model, thereby confirming their crucial involvement in the onset of LUSC or LUAD.

Survival data from individuals diagnosed with LUSC and LUAD were assembled, and gene interactions linked were systematically identified. These identified interactions were subsequently integrated as explanatory variables within the Cox regression model. Our hypothesis asserts that these gene interactions would demonstrate statistical significance in the model, thereby supporting their involvement in the onset of LUSC or LUAD.

For a comprehensive and in-depth exploration, we invite you to examine the associated GitHub repository (https://github.com/arriagajorge/Lung-cancer-multiomics/tree/master/Cox%20analisys).

## 3 Results and discussion

### 3.1 Representation of genetic interactions in LUAD and LUSC networks by categories and types of relationships

Our primary objective was to determine whether relationships between biological functions enriched through different sets of features in various datasets, when enriched multiple times, shared common underlying features and interactions. We observed that certain functions appeared frequently among the co-selected features in our analyses. Typically, functions that share common features can be identified through existing annotation databases. However, we aimed to go beyond these databases by leveraging our multi-omic integration strategy. This approach allowed us to uncover cross-linking patterns that might connect seemingly independent functions across different layers of biological data. Basically, we sought to understand how seemingly unrelated functions might be linked through shared patterns of variation in multi-omic data. To investigate these potential connections, we constructed networks based on mutual information (MI). MI is a measure that quantifies the degree of dependence or information shared between two variables, in our case, features and functions. These MI networks underwent a rigorous filtering process, in which we retained only the interactions that had a high probability of being regulatory. To determine the appropriate threshold for this filtering, we used MI values associated with known regulatory interactions. Specifically, we selected the median MI value as the minimum threshold required for considering an edge in the network as potentially indicative of regulatory relationships.

The network components we generated in this manner, provided a visual representation of the relationships between features and functions. These network components included features that had been annotated as participants in specific functions. The underlying assumption in our approach was that features selected through co-selection, which displayed correlated patterns with functional features, might also be involved in the regulation of these functions. The network includes nodes representing miRNAs, CpGs and transcripts that code for transcription factors. The threshold we applied during filtering aimed to exclude interactions primarily driven by simple co-variation while retaining those interactions that were more likely to have biological significance and serve as indicators of potential regulatory interactions within the network. In the Cytoscape visualizations presented, we adopted a visual encoding scheme that indicate important insights into the nature of interactions within the network. Specifically, activation interactions were depicted using bold lines, while functional associations were denoted with dashed lines. Subtle connections between genes, characterized by their relatively lower functional relevance or minor influence within the context of lung cancer, were illustrated by slender lines. Such linkages may signify indirect connections or associations of less pronounced significance; however, they still bear relevance within the broader network context.

The selection of this representation is supported by the high quality and reliability of the data from TCGA, the presence of attributes that describe specific relationships, and their biological relevance to lung cancer. In the following sections, we will present and discuss relevant findings, following a systematic approach. First, we will explore the data from LUAD, and subsequently, we will delve into the data from LUSC.

### 3.2 Expression of miR-125b-1, miR-125b-2 and miR-199a-2 is associated to LUAD development

MiR-125b-1 is produced by the long non-coding RNA (lncRNA) MIR100HG, its overexpression has been associated with oncogenic events, including abnormalities such as chromosomal translocation (t (2; 11) (p21; q32)). However, there is currently no evidence linking this miRNA to the development of NSCLC (Wang et al., 2020; Ashton et al., 2022). Whereas, it has been reported miR-125b-2 is generated from the miRNA cluster miR-99a/let-7c/miR-125b-2, located on chromosome 21. Studies suggest that inhibiting its expression may lead to a loss of differentiation in lung epithelial cells (Lee et al., 2005). On the other hand, the miR-199 family is a highly conserved group of miRNAs consisting of two members, miR-199a and miR-199b. Currently, two types of pre-miRNAs have been identified for miR-199a: pre-miR-199a–1 and pre-miR-199a-2. As a crucial member of the miRNA family, miR-199a has been implicated in various types of tumors, acting as either a suppressor or a promotor (Yang et al., 2021). In the context of NSCLC, miR-199a has been found to be significantly downregulated compared to normal tissue. Previous studies have consistently reported miR-199a as a tumor suppressor in NSCLC, and its reduced expression has been attributed to methylation abnormalities (Meng et al., 2022).

Drago et al., (Drago-García et al., 2017), previously utilized mutual information (MI) to study mir-199, constructing networks using data from both tumor and control tissues. Their findings indicated that this miRNA plays a crucial role in the transcriptional dynamics of breast cancer as well as normal tissue. However, as of now, gene expression networks have not been employed to explain the complexities of the development and (co)expression patterns of this particular miRNA in the context of LUAD.

**FIGURE 2:**
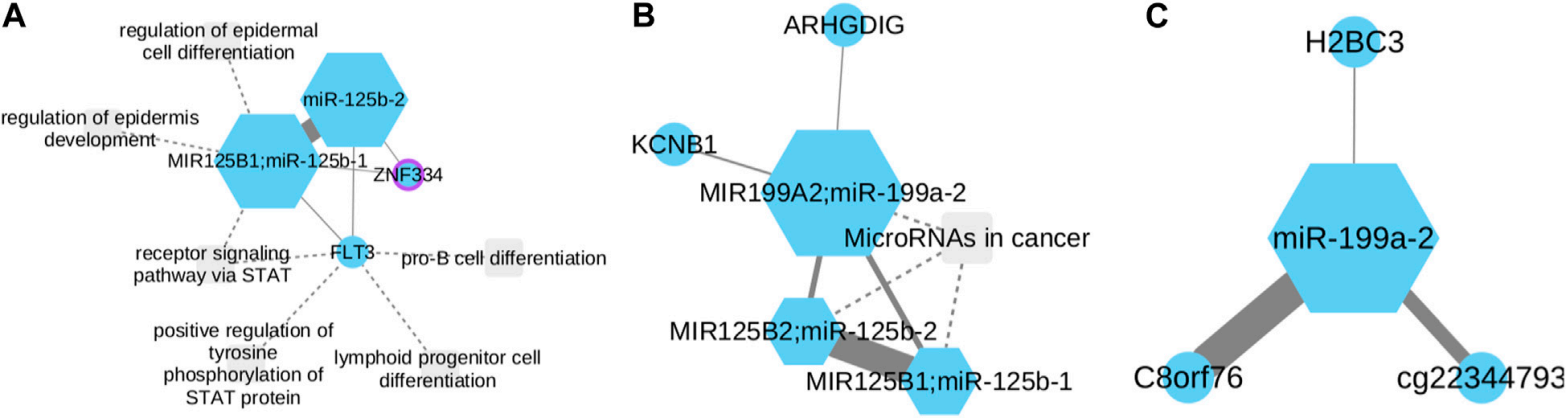
miR-125b-1, miR-125b-2 and miR-199a-2 functional subnetworks in LUAD. Circles represent CpGs, while squares symbolize transcripts. CpGs are identified with the gene symbol they affect; otherwise, the probe ID is used. Node size corresponds to its degree, with purple borders denoting proteins as transcription factors. Links’ strength is measured by mutual information between nodes. **(A)** miR-125b-1 and miR-125b-2 show connections with miR-199a-2. **(B,C)** MiR-199a is significantly downregulated in NSCLC and known as a tumor suppressor. The reciprocal relationship between miR-199a-2 and C8orf76 suggests unexplored oncogenic activities.

The generated networks highlight the presence of connections between miR-125b-1 and miR-125–2 (Figures 2A). Additionally, a relationship is observed involving miR-125b-1, miR-125b-2, and miR-199a-2 (Figures 2B). Furthermore, an independent network is formed by miR-199a-2 (Figures 2C). Regarding Figures 2A network, it is evident that both miRNAs exhibit minimal connections between two genes: FLT3 and ZNF334. FLT3 has been identified as being overexpressed in hematologic malignancies, such as acute myelogenous leukemia (Gilliland and Griffin, 2002). On the other hand, upregulation of ZNF334 has been associated with hepatocellular carcinoma (HCC), and triple-negative breast cancer (TNBC) (Yang et al., 2023). This limited association suggests that it is necessary to do an exhaustive research in FLT3 and ZNF334 properties related to NSCLC and re-evaluate its impact in the disease development. In the miR-125b-1; miR-125b-2; miR-199a-2 network, it is evident that the connection between the two first and mir-199a-2 is minimal. This observation aligns with existing information regarding the activity of these miRNAs in NSCLC. The methylated state of miR-199a-2 in tumor tissue, along with the overexpression of miR-125b-1 and miR-125b-2, supports this finding.

Furthermore, a connection between miR-199a-2 and two other genes, KCNB1 and ARHGDIG, can be observed. KCNB1 has been linked to colorectal and gastric cancer (Farah et al., 2020) while ARHGDIG (Rho GDP dissociation inhibitor gamma) is associated with vasopressin-related water reabsorption (Deckers et al., 2017). However, the information regarding their involvement in NSCLC remains unclear.

A significant correlation was observed in Figures 2C between mir-199a-2 and the C8orf76 gene. Notably, recent studies have demonstrated that silencing C8orf76 expression can effectively inhibit lung metastasis (Wang et al., 2019). This finding leads us to hypothesize that there is a reciprocal relationship between these two biomarkers in terms of their oncogenic activities that have not been explored yet.

To assess the potential clinical implications arising from the interaction among mir-125-b1, mir-125-b2, and mir-199-a2, a comprehensive examination was undertaken employing a Cox proportional hazards model. Upon meticulous adjustment of the Cox model, outcomes revealed statistically significant positive coefficients for mir-125-b1 and mir-125-b2 genes, with associated *p*-values of 0.029 and 0.016, respectively. These discerned associations imply a substantive correlation with patient survival within the context of lung cancer, suggesting that alterations in the expression or activity of mir-125-b1 or mir-125-b2 may have a considerable influence on patient outcomes. It is pertinent to note, that the individual microRNA, mir-199-a2, did not attain statistical significance (*p*-value = 0.181). Despite the lack of individual significance for mir-199-a2, its inclusion proves to be crucial for the observed significance attributed to the mir-125-b1 and mir-125-b2 genes. This is evident in the loss of statistical significance when the model incorporates mir-125-b1 and mir-125-b2 genes alone, with *p*-values of 0.18 and 0.17, respectively. This underscores the interdependence of mir-199-a2 in conjunction with mir-125-b1 and mir-125-b2 in influencing the survival dynamics in LUAD.

### 3.3 CAPN2 is strongly related to potential GPR27-regulation in LUAD

Calpains (CAPNs) constitute a family of cytosolic cysteine proteases activated by calcium (Zhang et al., 2018). Among the various isoforms, Calpain-2 (CAPN2) is recognized for its crucial involvement in biological processes, including cell migration, cytoskeletal remodeling, signal transduction and cell motility. Recent investigations conducted by Xu et al. (2019) showed that CAPN2 is upregulated in NSCLC and is correlated with a poor 5 year survival rate. Furthermore it has been proposed that inhibiting CAPN2 promotes apoptosis and inhibits proliferation of NSCLC.

**FIGURE 3:**
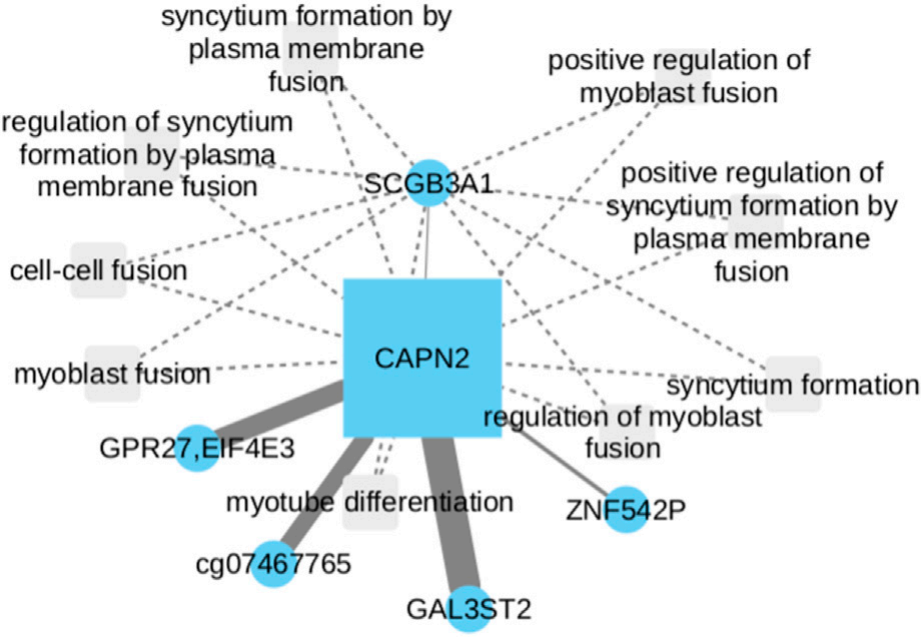
CAPN2 upregulation in NSCLC, linked to poor survival, involves cell migration and motility. While direct evidence is lacking for GAL3ST2 and GPR27 in NSCLC development, their presence with CAPN2 suggests GPR27 potential regulatory role in LUAD.

Our work revealed intriguing associations between CAPN2 and the GAL3ST2 and GPR27 genes (Figure 3). GAL3ST2 upregulation has been linked to robust expression in metastatic breast cancer tumors and prostate cancer (Guerra et al., 2015; Qin et al., 2016). On the other hand, the interactions of CAPN2 with GAL3ST2 and GPR27 as potential LUSC biomarkers, have been associated with methylation of the 3p11-p14 promoter region, a phenomenon observed in epithelial and cervical cancer (Lando et al., 2015). There is no currently direct evidence suggesting that GAL3ST2 or GPR27 are involved in NSCLC development. However, their presence in the network led us to consider the possibility of GPR27 potential involvement in regulatory processes within LUAD. Further investigations need to be done to elucidate the precise roles of GAL3ST2 and GPR27 in the context of LUAD and their potential interactions within CAPN2.

Following adjustments to the Cox model, a statistically significant positive coefficient was discerned for the CAPN2 gene (*p*-value = 0.047), thereby signifying a substantive association with survival within the context of lung cancer. This implies that alterations in CAPN2 expression or activity may exert an influence on patient survival. It is essential to note, that the individual CpG nodes GAL3ST2 and GPR27 did not independently exhibit statistical significance (*p*-values of 0.132 and 0.116, respectively). Although the CpG nodes GAL3ST2 and GPR27 did not attain individual statistical significance, their inclusion proves to be essential for the observed significance concerning the CAPN2 gene. This is evident from the loss of significance when the model exclusively incorporates the CAPN2 gene (*p*-value >0.5).

### 3.4 PFN2 and TBL1XR1 are related to a potential LUSC-associated transcript

Profilin-2 (PFN2) belongs to the family of the actin-binding proteins, and its expression is commonly linked with the nervous system, playing a role in neurotransmitter exocytosis (Ling et al., 2021). Recent research has demonstrated that PFN2 upregulates the expression of Smad2 and Smad3 through an epigenetic mechanism. Additionally, this upregulation of PFN2 and Smad expression has been associated with an unfavorable prognosis of lung cancer patients (Tang et al., 2015). Transducin (*β*)-like 1 *X*-linked receptor 1 (TBL1XR1) has been implicated in high metastatic rates observed in breast, gastric, and stomach cancers. Moreover, its overexpression in NSCLC cell lines has been shown to drive cell survival, proliferation, and metastases (Zhang et al., 2020).

**FIGURE 4:**
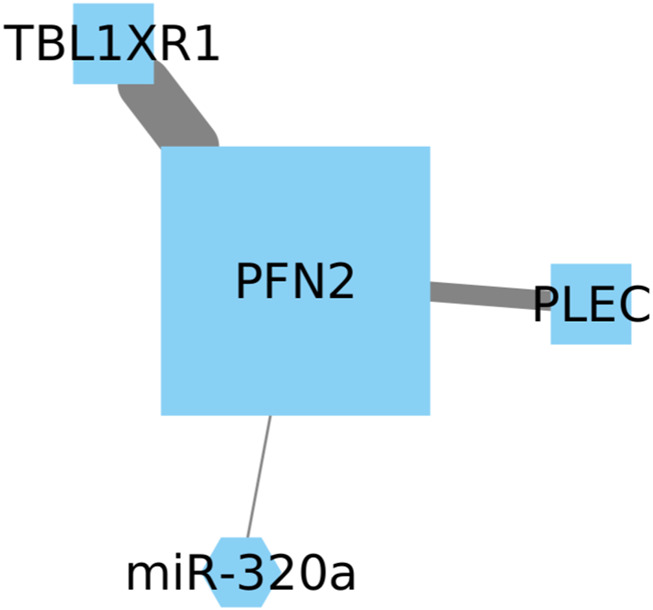
Upregulation of PFN2 and TBL1XR1 is linked to an unfavorable prognosis in LUSC. We also identify PLEC as a significant gene with potential correlation, but its relationship with PFN2 and TBL1XR1 remains unexplored in NSCLC.

While both genes show high expression in lung cancer patients with a poor prognosis, we have identified another significant gene that may potentially correlate with the expression of these genes (Figure 4). Plectin (PLEC) is a protein known to be involved in binding and modulating the proto-oncogene tyrosine-protein kinase FER and the energy-controlling AMP-activated protein kinase (Wesley et al., 2021). However, the relationship between PLEC and PFN2 or TBL1R1 remains unexplored, and its impact in NSCLC has been scarcely studied, with its most recent report being in squamous cell lineages (Nie et al., 2023).

The Cox model revealed statistically significant positive coefficients for the TBL1XR1 gene and the interaction between PLEC and TBL1XR1 genes. The associated *p*-values of 0.029 underscore the substantive nature of these associations, particularly within the landscape of lung cancer survival. These findings suggest that variations in the expression or activity of TBL1XR1 and PLEC may have a considerable influence on patient survival. It is essential to emphasize that the individual CpG node PFN2 did not achieve statistical significance (*p*-value = 0.852). While the independent impact of PFN2 did not reach statistical significance, its indispensability becomes apparent considering the observed significance linked to the PLEC gene and TBLXR1. This is highlighted by the decrease in statistical significance when the model involves only the PLEC and TBLXR1 genes, yielding *p*-values of 0.11 and 0.33, respectively.

### 3.5 Inhibition of LRP1 might be related with TBXAS1 increment in LUSC

The low-density lipoprotein receptor-related protein 1 (LRP1), is a large transmembrane receptor (abundantly produced by fibroblasts). In both, LUAD and LUSC, it has been demonstrated that LRP1 decreases its expression compared to healthy tissues, while in other tissues like brain cancers its expression increases, suggesting that LRP1 plays a role in glioma growth (Lopes et al., 1994). Thromboxane A synthase 1 (TBXAS1) is known to play functional roles in processes such as neoplastic transformation, including cell motility and invasion, proliferation, and therapeutic resistance (Ashton et al., 2022).

**FIGURE 5:**
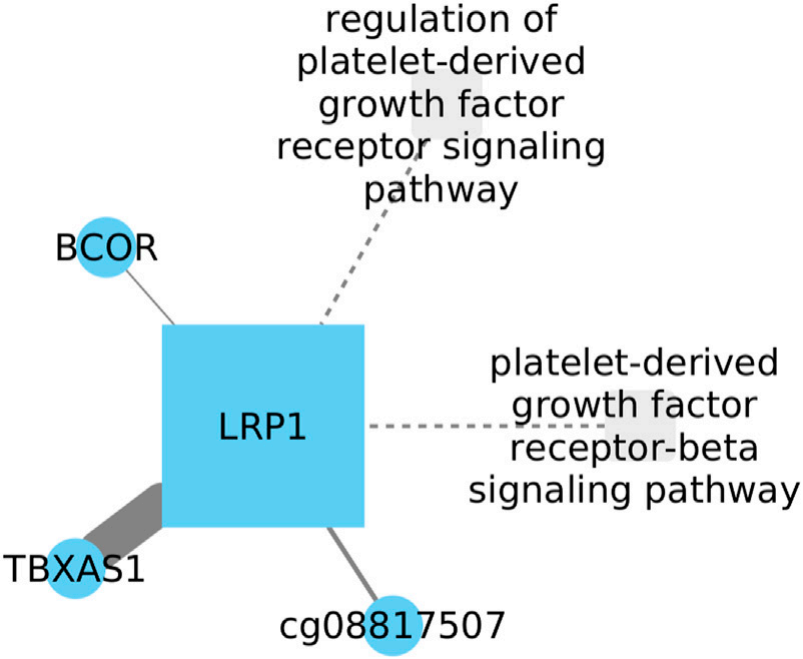
TBXAS1 shows a link to LRP1 in cancer gene expression regulation. While TBXAS1 potential as a lung cancer biomarker is unclear, LRP1 downregulates it during lung cancer development. The exact influence of TBXAS1 on its expression in LUSC remains uncertain.

Our research has revealed a relationship between TBXAS1 and LPR1 in the regulation of gene expression in cancer (Figure 5). Although TBXAS1 has not been clearly established as a potential biomarker for lung cancer, it is known that in lung cancer development, LRP1 downregulates its expression. However, the direction of TBXAS1 influence on its expression during lung cancer development remains uncertain. Interestingly, elevated levels of TBXAS1 have been observed in breast cancer samples with a poor prognosis (Watkins et al., 2005), suggesting a potential parallel in lung cancer. These findings lead us to speculate that TBXAS1 might also be associated with an unfavorable outcome in lung cancer.

Investigating the clinical implications of LRP1, TBXAS1, and BCOR using the Cox model we observed interactions among these genes did not demonstrate statistical significance. However, it is remarkable that the interaction involving LRP1 approached significance, presenting a *p*-value of 0.057.

Investigating LRP1, TBXAS1, and BCOR using the Cox model we observed interactions among these genes did not demonstrate statistical significance. However, it is important to highlight that the interaction related to LRP1 came close to significance (*p*-value of 0.057). This subtle but suggestive result encourages additional exploration of the complex relationships between these genes, emphasizing the necessity for ongoing research to uncover their potential roles in clinical settings.

### 3.6 Ribosomal proteins S6 and S18 may be implicated in LUSC development

Ribosomal protein S6 (RPS6), a 40S ribosomal subunit, has been extensively investigated and is believed to play a significant role in stimulating protein translation. Recent data indicate that phosphorylated RPS6 might serve as a potential tumoral biomarker (Knoll et al., 2016). In the context of NSCLC, RPS6 has been observed to be overexpressed, and it is hypothesized that its downregulation could inhibit tumoral tissue growth by inducing G0-G1 cell cycle arrest (Chen et al., 2014). SRC Kinase Signaling Inhibitor 1 (SRCIN1) is a protein-coding gene that Ye et al. (2016) proposed to be involved in cell proliferation during NSCLC development when silenced.

**FIGURE 6:**
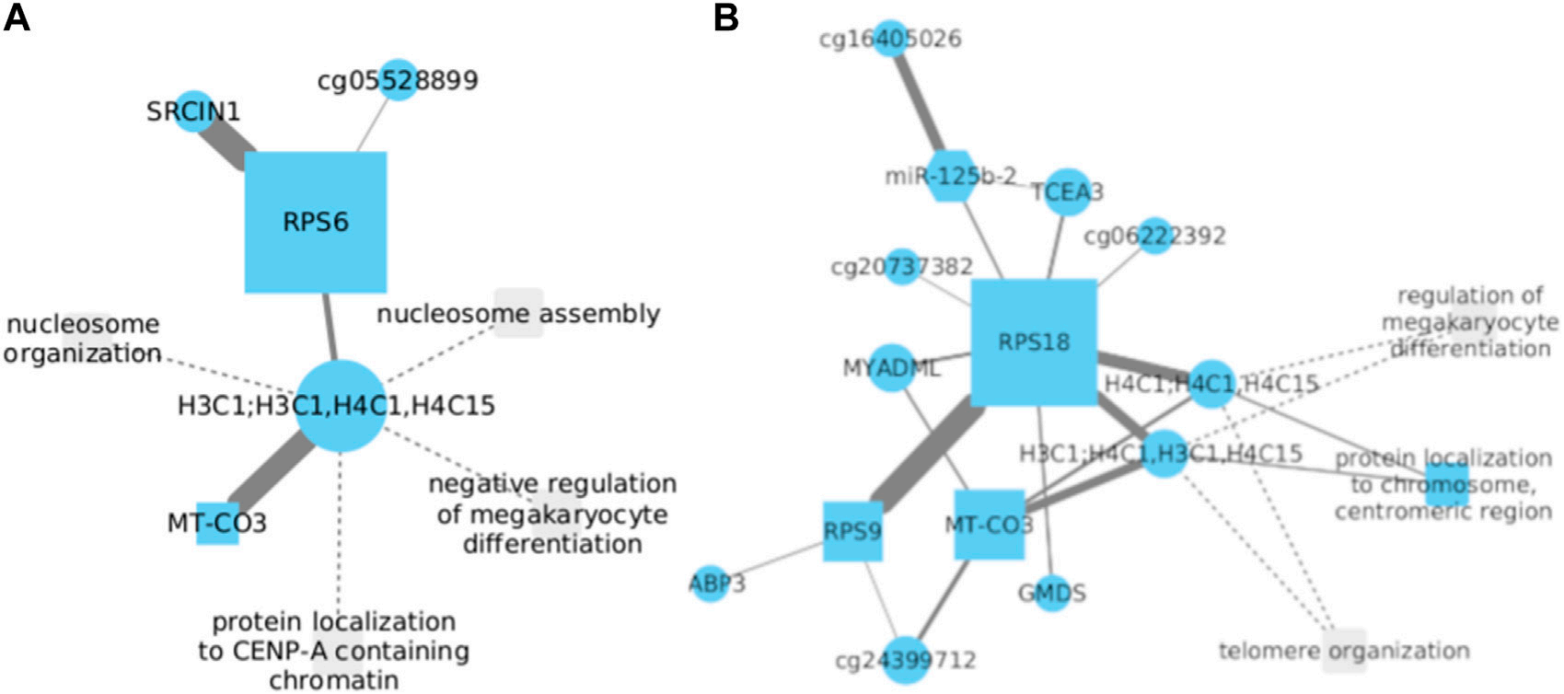
**(A)** Significant correlation between RPS6 and SRCIN1 genes in NSCLC was observed. Silencing of SRCIN1 is proposed to promote cell proliferation during NSCLC development. Additionally, RPS6 and SRCIN1 show strong interconnectedness in tumorigenesis. **(B)** In LUSC, RPS18 and RPS9 demonstrate a direct association with opposing functions, implying an inverse relationship. Overexpression of RPS18 may favorably inhibit RPS9, accentuating its role in cancer.

The analysis of gene expression data from NSCLC demonstrated a strong correlation between RPS6 and SRCIN1 (Figures 6A), implying a potential interconnected relationship in tumorigenesis. Based on the data we have acquired, it is hypothesized that a positive correlation should exist between these two genes. This would entail mutual upregulation during the progression of LUSC development. To gain a comprehensive understanding, further investigations are needed to elucidate the precise mechanism by which these two genes interact and potentially contribute to the development and progression of NSCLC.

Furthermore, an association was identified between RPS6 and the core histone proteins H3C1, H4C1, and H4C15. Nonetheless, this relationship does not exhibit the same level of significance observed with SRCIN1. Particularly noteworthy is the correlation between core histones and MT-CO3. This gene, when dysregulated, has been investigated for its involvement in cellular metabolic alterations and its potential role in facilitating the transition of normal cells into malignant ones. However, the activity of MT-CO3 in NSCLC has not been documented thus far. To evaluate the potential clinical ramifications of RPS6 interactions with H4C1 and H3C1, the Cox model showed statistical significance with positive coefficients for the H4C1 gene, as well as the interactions between H4C1 with both RPS6 and H3C1 genes. Additionally, a significant interaction between H3C1 and RPS6 genes was noted. The associated *p*-values for these interactions were 0.0383, 0.0234, 0.0202, and 0.0155, respectively. These findings underscore a significant correlation with survival within the context of lung cancer. They suggest that modifications in the expression or activity of H4C1, H3C1, and RPS6 may manifest a significant impact on patient survival.

Comparably, overexpression of Ribosomal Protein S18 (RPS18) has been linked to tumoral growth, particularly in esophageal and breast cancer (Riehle et al., 2010; Xu et al., 2022). However, there is no existing report that establishes a correlation between RPS18 expression and LUSC. On a different note, Ribosomal Protein S9 (RPS9) has been associated with the inhibition of cell proliferation (Yang et al., 2021).

Our findings reveal a direct association between RPS18 and RPS9 in their expression patterns during the progression of LUSC (Figures 6B). Notably, considering their opposing functions, where RPS9 expression inhibits cell proliferation in lung cancer development while RPS18 is significantly overexpressed in cancer, we propose an inverse relationship between them. This put forward that the overexpression of one gene implies the inhibition of the other, with RPS18 being the favored gene in this particular relationship.

Conversely, we have identified a robust association between RPS18 and the core histone proteins H3C1, H4C1, and H4C15. This observation leads us to propose that, despite the limited correlation observed between these histones and ribosomal protein S6 (RPS6), their connection with RPS18 provides a more substantial basis to speculate their potential involvement in the progression of NSCLC and LUSC. We have also observed the formation of connections between the central node, RPS18, and other genes. Although these associations exhibit a lower level of affinity, it remains uncertain whether these genes contribute to the development of LUSC or NSCLC, as literature has not reported any information concerning these genes’ roles.

### 3.7 EIF4G1 and related genes in LUSC tumorigenesis

Eukaryotic Translation Initiation Factor 4 Gamma 1 (EIF4G1) has been linked to tumorigenesis and tumor progression. In NSCLC, its expression has been observed to be significantly higher in tumor tissues compared to normal lung tissue (Cao et al., 2016; Del Valle et al., 2021).

**FIGURE 7:**
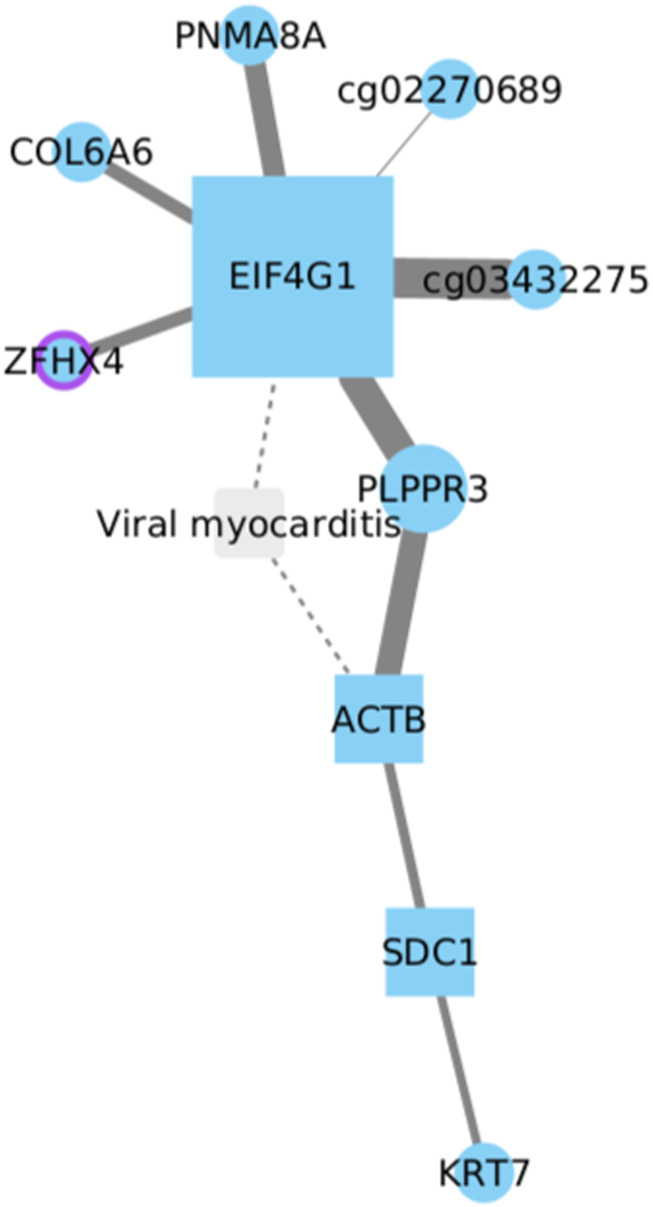
EIF4G1 directly interacts with six genes, including ZFHX4 and COL6A6, associated with LUAD. PLPPR3, unrelated to lung cancer, links with ACT, SDC1, and KRT1, previously reported to be implicated in lung cancer. PNMA8A and PLPPR3 also show implications in cancer development, however, their involvement in lung cancer is still unknown.

The evidence reported suggests that in LUSC, EIF4G1 directly interacts with six other genes (Figure 7). Among these, only two genes have been studied and associated with NSCLC, specifically in LUAD and not LUSC. These genes are ZFHX4, an overexpressed gene in LUAD linked to more aggressive disease characteristics and a poor prognosis (Xia et al., 2019), and COL6A6, considered a tumor suppressor and therapeutic target in LUAD (Ma et al., 2021). However, among the six EIF4G1-related genes, PLPPR3, which is not typically associated with lung cancer, stands out as it is linked to three other genes: ACT, SDC1, and KRT1. Each of these genes has been previously implicated in lung cancer development (Guo et al., 2013; Koren et al., 2015; Götte and Kovalszky, 2018). Still, it remains unclear whether they share any relationship with EIF4G1 expression during the course of the disease, particularly in LUSC development.

Based on current information, both PNMA8A and PLPPR3 are implicated in cancer development. In the case of PNMA8A, this gene has been associated with apoptosis and colorectal cancer (Yang et al., 2022). On the other hand, PLPPR3 has been less frequently linked to cancer processes and is primarily associated with cardiovascular and neurodegenerative diseases (Yurikova et al., 2019). Considering the potential involvement of these unrelated genes, PNMA8A and PLPPR3 should not be disregarded in terms of their potential implications in NSCLC, particularly in LUSC development.

Upon adjustment of the Cox model, outcomes manifest statistically significant positive coefficients for the ZFHX4 gene and the cooperative interplay between ZFHX4 and COL6A6 genes, resulting in *p*-values of 0.041 and 0.043, respectively. These discerned patterns imply a correlation with survival. Such observations suggest that alterations in the expression or functionality of ZFHX4 and COL6A6 genes could exert a tangible influence on patient survival. It is crucial to note that the individual gene node EIF4G1 did not exhibit statistical significance, as evidenced by a *p*-value of 0.65.

While the individual significance of EIF4G1 might not be remarkable, its indispensability becomes apparent in the context of the observed significance associated with the ZFHX4 gene and COL6A6. This is evident through the attenuation of significance when the model involves the ZFHX4 and COL6A6 genes, resulting in *p*-values of 0.26 and 0.69, respectively.

### 3.8 EEF2, LRP1, TRIO and DST might have mutual interaction in LUSC

**FIGURE 8:**
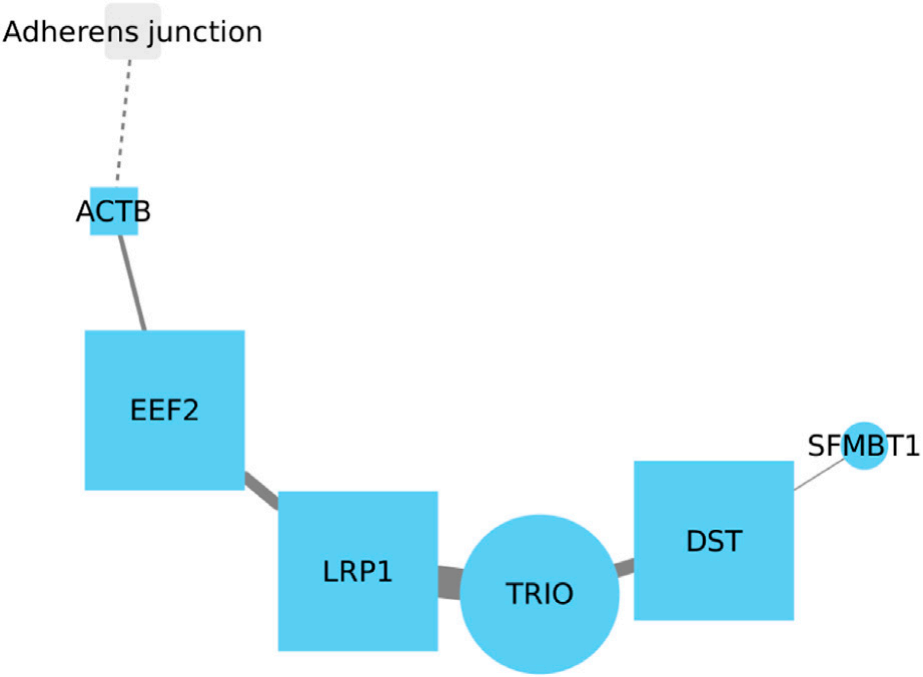
Further investigation into the interactions of EEF2, TRIO, and DST with LRP1 is crucial, given LRP1 downregulation in lung cancer. Understanding these connections may explain their roles in lung cancer. ACTB and SFMBT1 show weak associations in the network, with SFMBT1 lacking reported links to lung cancer.

There is no reported relationship between EEF2 and LRP1. However, their roles in lung cancer appear to be opposite. Active EEF2 promotes tumor growth in lung cancer, whereas active LRP1 decreases its activity in lung tumor tissue (Meng et al., 2011; Oji et al., 2014). Whereas the expression of DST is similar to EEF2, being overexpressed in lung cancer tissues. Recent reports link DST to the development of LUAD, but there is no information on whether this gene may be related to the development of LUSC. Notably, TRIO (The Triple Functional Domain gene) is the only gene in this network (Figure 8) that has been reported to be associated with LUSC (Garnis et al., 2005). Based on the previously reported and current information, it is strongly suggested that the relationship among these four genes warrants further investigation, particularly the interactions of EEF2, TRIO, and DST in relation to LRP1, which is the only gene in this set which is downregulated in lung cancer development. Understanding the intricate connections between these genes could provide valuable insights into their potential roles and contributions in the context of lung cancer.

While ACTB and SFMBT1 are included in this gene interaction network, it is noteworthy that neither node exhibits a robust relationship with the rest of the nodes, particularly SFMBT1, which has not been reported in relation to lung cancer (Pan et al., 2022). Although ACTB has been previously implicated in tumor development in lung cancer, its connection with other genes in this context requires further examination and understanding.

Following adjustment for relevant covariates, our analysis revealed statistically significant positive coefficients for the interactions involving LRP1 with both DST and EEF2 genes, as well as the interaction among the three genes EEF2, LRP1, and DST. The associated *p*-values for these interactions were determined to be 0.043, 0.032, and 0.021, respectively. These statistical outcomes underscore a notable association with survival within the context of lung cancer. Our findings suggest that alterations in the expression or activity of EEF2, LRP1, and DST may have a substantial influence on patient survival outcomes.

### 3.9 Comparison of central and topological network measures

Through meticulous examination, we describe centrality measures, Average Shortest Path Length (ASPL) (Mao and Zhang, 2013), Betweenness Centrality (BC) (Dolev et al., 2010), and Closeness Centrality (CC) (Borgatti, 2005), along with topological metrics like Neighbourhood Connectivity (NC) (Estrada and Bodin, 2008) and Topological Coefficients (TC) (Assenov et al., 2008), providing valuable information on efficiency, connectivity and collaborative structures in the LUAD and LUSC networks.

We individually assessed the LUAD networks for each parameter. The resulting means for each parameter revealed a substantial similarity between the LUAD networks, as indicated in Table 1. Following this individual assessment, we conducted a comprehensive evaluation of the data by averaging the means derived from each network. A significantly low ASPL (1.76) indicated efficient global connectivity. For our purposes, this could be indicative of co-expression patterns, where genes tend to be expressed together in response to certain biological processes or conditions during LUAD development. The average BC of 0.14 is relatively high, leading us to suggest that core genes in each network may be regulators influencing the flow of signals or information in the gene expression network. The average CC of 0.60 suggests that nodes can efficiently communicate with each other within the networks. In the context of LUAD, data suggests that the core genes of the networks may be potential candidates for therapeutic targets due to their central roles in the gene expression networks. Noteworthy NC (5.82) and TC (0.27), imply that groups of genes within the networks are co-regulated or participate in similar biological functions, as suggested in the previous sections. Relatively, low standard deviations across metrics affirmed the coherence and robustness of the LUAD network structure, providing valuable insights into its functional organization.

**TABLE 1:** Comparison of LUAD networks. Mir199a2 shows a compact network with efficient information transmission, as indicated by its shorter average path length (1.50). This structural feature aligns with the known regulatory functions of microRNAs, which often exert control over multiple target genes. The elevated betweenness centrality (0.25) of mir199a2 suggests its crucial role in orchestrating communication within the network, potentially influencing pathways associated with cancer progression or other biological processes. On the other hand, CAPN2, displays a network with a longer average path length (1.781) but higher neighborhood connectivity (11.299) and topological coefficient (0.543). These characteristics point to a more densely interconnected and clustered network for CAPN2. In a biological context, this might indicate that CAPN2 operates in a modular and tightly regulated manner, potentially participating in distinct cellular processes or signaling cascades. The lower betweenness centrality of CAPN2 (0.060) suggests a more distributed influence within its network, consistent with its role as a protease involved in various cellular functions. Furthermore, the network properties of mir125b-mir199a2 and mir125b exhibit intermediary characteristics, with values falling between those of mir199a2 and CAPN2. This suggests potential collaborative interactions between mir125b and mir199a2 or unique regulatory roles for mir125b in the context of its network architecture.

	CAPN2 net	mir125b-mir199a2 net	mir199a2 net	mir125b net
ASPL	1.781	1.933	1.50	1.821
BC	0.060	0.117	0.25	0.137
CC	0.580	0.539	0.70	0.573
NC	11.299	5.000	2.50	4.500
TC	0.543	0.255	0.00	0.298

For the LUSC data, we conducted a parallel analysis, individually analyzing parameters. The means calculated for each parameter disclosed a marked similarity between the LUSC networks, such as LUAD networks and is delineated in Table 2. Subsequently, the means of each LUSC network were averaged and compared with the LUAD mean (Table 3). The examination of the LUSC networks revealed an ASPL of 2.11, suggesting that the network is organized in a way that facilitates rapid and effective communication among genes in the networks. The average BC of 0.18 indicates that the core genes may serve as potential network-based biomarkers for LUSC. Their central roles in the network suggest their importance in the context of disease, and alterations in their expression or activity could have significant implications for cancer progression as we have suggested in the previous sections. The average CC of 0.52 suggest that functionally related genes are tightly connected. This reflects the presence of cohesive functional modules or pathways associated with LUSC. Additionally, NC was 3.92, meaning that if one gene in the network is disrupted, connectivity between its neighbours can help maintain the overall stability of the network, while the average TC of 0.24 suggested close collaborations between nodes, reaffirming that the genes and CpG sites represented in the networks are involved in similar or complementary biological functions. The low standard deviation in the Average Shortest Path Length and Closeness Centrality we obtained suggests stability in network efficiency.

**TABLE 2:** Comparison of LUSC networks. Notably, PFN2 shorter paths (1.500) and higher closeness centrality (0.695) imply a potential central role in facilitating rapid and efficient information exchange, positioning it as a key player in its network. EEF2 stands out with the highest betweenness centrality (0.333), suggesting it acts as a critical mediator in connecting different parts of its network. In contrast, LRP1 and PFN2 exhibit comparable betweenness centrality values (0.167), emphasizing their potential influence on information transfer within their respective networks. These findings have implications for understanding the regulatory dynamics of these transcripts, with higher betweenness centrality indicating a greater influence on network connectivity. The neighborhood connectivity and topological coefficient values provide insights into the local and global organization of the networks. For instance, RPS18’s high neighborhood connectivity (5.039) suggests extensive interactions with its neighboring nodes, possibly indicative of its involvement in tightly coordinated cellular processes. PFN2’s elevated topological coefficient (0.400) suggests the formation of local clusters, indicating potential functional modules within its network.

	EIF4G1 net	LRP1 net	PFN2 net	RPS18 net	RPS6 net	EEF2 net
ASPL	2.436	1.667	1.500	2.331	2.056	2.667
BC	0.160	0.167	0.167	0.089	0.151	0.333
CC	0.431	0.630	0.695	0.444	0.508	0.392
NC	4.753	4.333	3.100	5.039	4.444	1.857
TC	0.221	0.000	0.400	0.398	0.056	0.357

**TABLE 3:** Comparing both networks. LUAD exhibits a more compact network with a shorter average path length compared to LUSC, suggesting a more efficient information flow. The lower betweenness centrality in LUAD implies a decentralized information mediation system, contrasting with LUSC higher centrality. Higher closeness centrality in LUAD indicates nodes are closer on average, potentially influencing network cohesion.The analysis extends to neighborhood connectivity, where LUAD nodes display more interconnections than LUSC, suggesting a heightened network complexity in LUAD. Furthermore, the topological coefficient is higher in LUAD, reflecting increased local clustering, potentially signifying distinct regulatory modules.

	LUAD	LUSC
ASPL	1.759	2.109
BC	0.141	0.178
CC	0.598	0.517
NC	5.825	3.921
TC	0.274	0.239

### 3.10 CpG sites function as promoters

Analysis of CpG sites was conducted to discern their functional roles within the gene expression networks. Promoter data for this analysis were sourced from the UCSC Genome Browser database (Karolchik et al., 2003) through the TxDb.Hsapiens.UCSC.hg38.knownGene library, with detailed information available in the GitHub repository. To identify enhancers, data were directly retrieved from the ENCODE page, specifically focusing on enhancer data related to human lung tissue, as released by Jesse Enfreitz’s lab at Stanford. A total of 276,905 promoters and 2,397,507 enhancers were associated with genes of lung tissue from the UCSC database. Genomic coordinates for these regulatory elements were generated through BED files, utilizing information from the ENCODE database. The intersection of these sets revealed 2,674,412 unique elements, which were subsequently classified as promoters or enhancers. The initial findings of this intersection are available at the Github repository, presenting information such as transcript identifier (ENST ID), genomic coordinates, element type (promoter or enhancer), and others. Following this, CpG sites were associated with the identified promoters and enhancers. Using data from the Ensembl database, transcript identifiers (ENST ID) were obtained for all CpG sites reported in this study.

Notably, all identified CpG sites were classified as promoters; no CpG sites with enhancer activity were observed. The consistent classification of all CpG sites as promoters suggests their potential involvement in transcription initiation and regulation processes (Yan et al., 2017). Supplementary Table S5 illustrates the association between CpG sites and regulatory elements. A merging of gene data with the promoter and enhancer database resulted in a comprehensive dataset containing detailed information on the genomic locations of regulatory elements associated with each gene (Supplementary Table S6). Lastly, distances between genes of interest and their associated CpG sites were calculated. These distances represent the difference in midpoint positions between the genes and their respective CpG sites and can be referred to in Supplementary Table S7.

## 4 Conclusion

High-throughput technologies have catalyzed a paradigm shift by enabling extensive genome-wide association studies and facilitating the examination of global transcriptomic profiles. The incorporation of multi-omics data into cancer research has led in a systems biology perspective, making use of the interconnections among various molecular characterizations, to name a few, Tian et al. (2022) developed of a classification approach for Non-Small Cell Lung Cancer (NSCLC), involving the integration of diverse multi-omics data to construct a comprehensive background interaction network. Yang et al. (2019) integrated data from various omics sources and utilized statistical tests to identify notable distinctions between cancerous and normal tissues.

In spite of that, the effort to fulfill a mechanistic comprehension continues to present an enduring challenge. In order to formulate exhaustive genomic and transcriptomic regulatory charts capable of summarize the complexity of lung cancer, it is imperative to scrutinize a multitude of gene expressions and high-dimensional genetic variations.

Our research methodology is distinctly characterized by its primary focus on functional analysis within lung cancer subtypes. Rather than emphasizing classification, this study prioritizes the understanding of functional implications arising from multi-omic interactions. To achieve this, we conduct subtype-specific analyses, delving into the complexities of multi-omic interactions within different lung cancer subtypes, specifically LUAD and LUSC. These analyses aim to unveil potential subtype-specific mechanisms, utilizing ARACNE network analysis method to scrutinize the intricate relationships between CpG sites, miRNA, and transcripts within these subtypes. By doing so, we shed light on regulatory and signaling pathways. Our study’s key objective is to assess the functional impact of multi-omic interactions. To achieve this, we employ methods such as functional enrichment analysis and pathway analysis to uncover the biological consequences and associations of these interactions. This unique approach offers a deeper understanding of the functional relevance of multi-omic data within the context of lung cancer.

Through SGCCA analysis, we successfully identified enriched functions in DNA methylation, transcript, and miRNA expression features that showed covariation. However, it is worth noting that SGCCA does have a drawback, mainly attributed to LASSO’s instability. To address this concern, we chose to retain only those features present in over 70% of subsamples, ensuring a more stable feature set (Ochoa and Hernández-Lemus, 2023). We favor the sparse method for its reliability in feature selection (Kang et al., 2013).

Taking into account the significant SGCCA findings, we conducted a thorough functional enrichment analysis using ClusterProfile, focusing on the non-zero loadings within the sparse matrix. This approach allowed us to gain deeper insights into the underlying biological mechanisms and potential pathways involved in our study. Comparing interest features with KEGG database pathways and Gene Ontology (GO) biological processes, we performed over-representation tests on features enriched exclusively in single datasets. To enhance understanding, we further clustered these uniquely enriched features based on their GO and KEGG classes, adding valuable insights to our research. Additionally, we performed a gene set enrichment analysis (GSEA) using transcript data to explore functions affected by differential expression. Leveraging the clusterProfiler package without a *p*-value cutoff, we obtained GSEA enrichment scores for functions overrepresented in the SGCCA results, thus enriching our understanding of the multiomic landscape.

Subsequently, the selected functions were visualized as networks for each cancer subtype (Supplementary Figure S3), facilitating the construction of potential regulatory models. This network-based approach enabled us to identify all features that co-varied with those responsible for functional enrichment, making more understandable the intricate relationships within this complex biological system. To estimate mutual information between node pairs, we considered CpG interactions and gene expression influenced by position overlap using microarray annotation data. For transcript analysis, we utilized the TFtargets package, while the multiMiR package was employed for miRNA investigation, thereby enhancing the depth and scope of our analysis.

To determine the threshold for regulatory interactions, we chose the median of MI (Mutual Information) values, mitigating the influence of dominant outliers and ensuring the robustness of our results. As MI values varied between different pair types, distinct thresholds were obtained for CpG-transcript, CpG-miRNA, TF-transcript-transcript, and miRNA-transcript edges, refining our assessment of the regulatory network. In cases where distributions showed no significant differences, we selected the lowest median MI as a single threshold, thereby accommodating more MI interactions in the final network. To enhance the robustness of our analysis, we employed the Kolmogorov-Smirnov test for a thorough comparison of MI value distributions among different edge types.

Our comprehensive analysis of gene networks across studies sheds light on intricate relationships within non-small cell lung cancer (NSCLC). The miRNA networks encompassing miR-125b-1, miR-125b-2, and miR-199a-2 hold potential implications for LUAD, urging further exploration into FLT3 and ZNF334 associations. The connections among CAPN2, GAL3ST2, and GPR27 genes open doors to intriguing NSCLC regulation prospects, in this study we observed the relation between the in LUAD type, necessitating deeper investigations into GPR27’s role. PFN2 and TBL1XR1 exhibit potential correlations with poor prognosis, and PLEC emerges as a promising connector, warranting in-depth examination. The intricate interplay of TBXAS1 and LRP1 influences cancer gene expression, while TBXAS1’s elevated levels in poor-prognosis breast cancer samples suggests a parallel lung cancer implications for LUSC. Simultaneously, the RPS6-SRCIN1 correlation propose interconnected tumorigenesis. The unique RPS18-RPS9 dynamic in LUSC progression points towards an inhibitory relationship favoring RPS18 overexpression. Finally, EIF4G1 displays diverse interactions in LUSC, linking with LUAD-related ZFHX4, COL6A6, and unusual PLPPR3 connections to lung cancer genes. The contrary functions of EEF2 and LRP1 in lung cancer, along with DST shared expression with EEF2, are evident. TRIO’s sole LUSC association highlights its relevance with EEF2, DST, and LRP1 interactions. Further research is vital for comprehending ACTB’s role and SFMBT1’s uncertain link. Within these genetic networks, previously unexplored relationships emerge, inviting dedicated investigation. These findings collectively emphasize the complex molecular web. Thus, exhaustive research is imperative to unveil the roles and interactions of these genes within the broader context of NSCLC development.

In conclusion, our study showcases the potential of integrating multi-omics data, including RNA-seq, miRNA-seq, and human methylation data, to unravel the intricate mechanisms of gene regulation. By illuminating the interplay between transcriptional and epigenetic processes, our work significantly contributes to a deeper comprehension of lung cancer. These valuable insights may pave the way for the development of innovative therapeutic strategies and diagnostic approaches in the context of Lung Adenocarcinoma and Lung Squamous Cell Carcinoma.

## Data Availability

Publicly available datasets were analyzed in this study. This data can be found here: https://portal.gdc.cancer.gov/projects/TCGA-LUSC. https://portal.gdc.cancer.gov/projects/TCGA-LUAD.
