# Supplementary material for: Functional impact of multi-omic interactions in lung cancer
Source: Front Genet. 2024 Feb 8;15:1282241. doi: 10.3389/fgene.2024.1282241 (PMC10881857; doi:10.3389/fgene.2024.1282241)
Supplement: Supplementary file 3 [file DataSheet1.PDF]

Supplementary Material

1 SUPPLEMENTARY DATA

2 SUPPLEMENTARY TABLES AND FIGURES

2.1 Tables

Table S1. Functional enrichment results for the gene ontology of biological processes for LUAD.

Table S2. Functional enrichment results for the gene ontology of biological processes for LUSC.

Table S3. Functional enrichment results for the KEGG pathways for LUAD.

Table S4. Functional enrichment results for the KEGG pathways for LUSC.

2.2 Figures

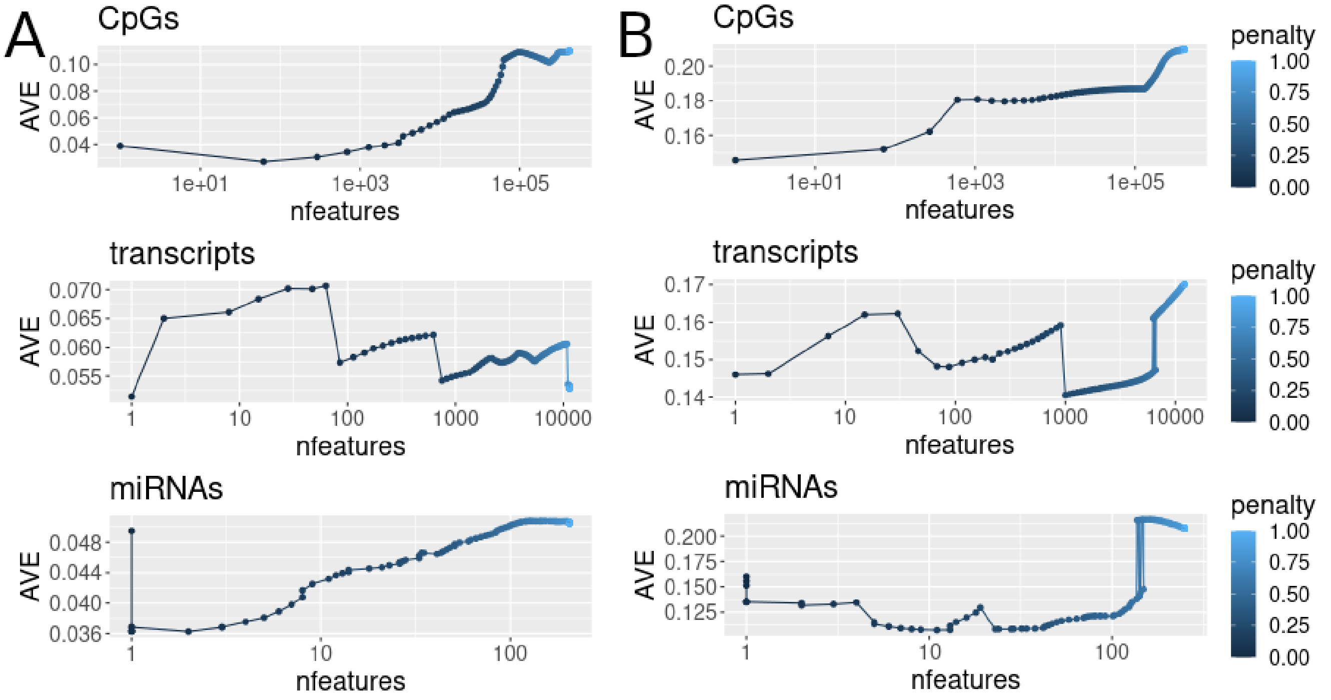

Figure S1. Selection of the sparsity parameters. A) LUAD. B) LUSC. Sparsity parameters were fitted by cross-validation with $k = 5$, testing values within [0.01,0.02,. . . ,0.98, 0.99]. We choose the dot before the largest change in slope, taking 0.01 for CpG sites, 0.02 for transcripts and 0.11 for microRNAS in LUSC type, while taking 0.30 for CpG sites, 0.01 for transcripts and 0.14 for microRNAS in LUAD type.

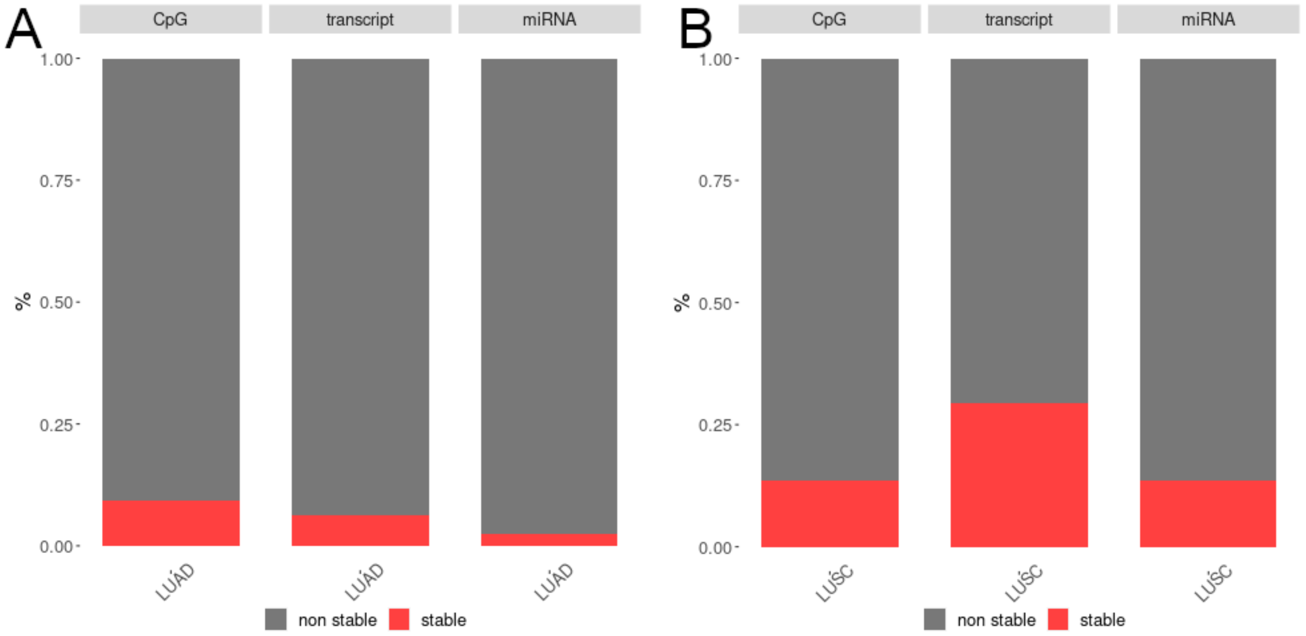

Figure S2. Proportion of stable features per omics and types of cancer. A) LUAD. B) LUSC.

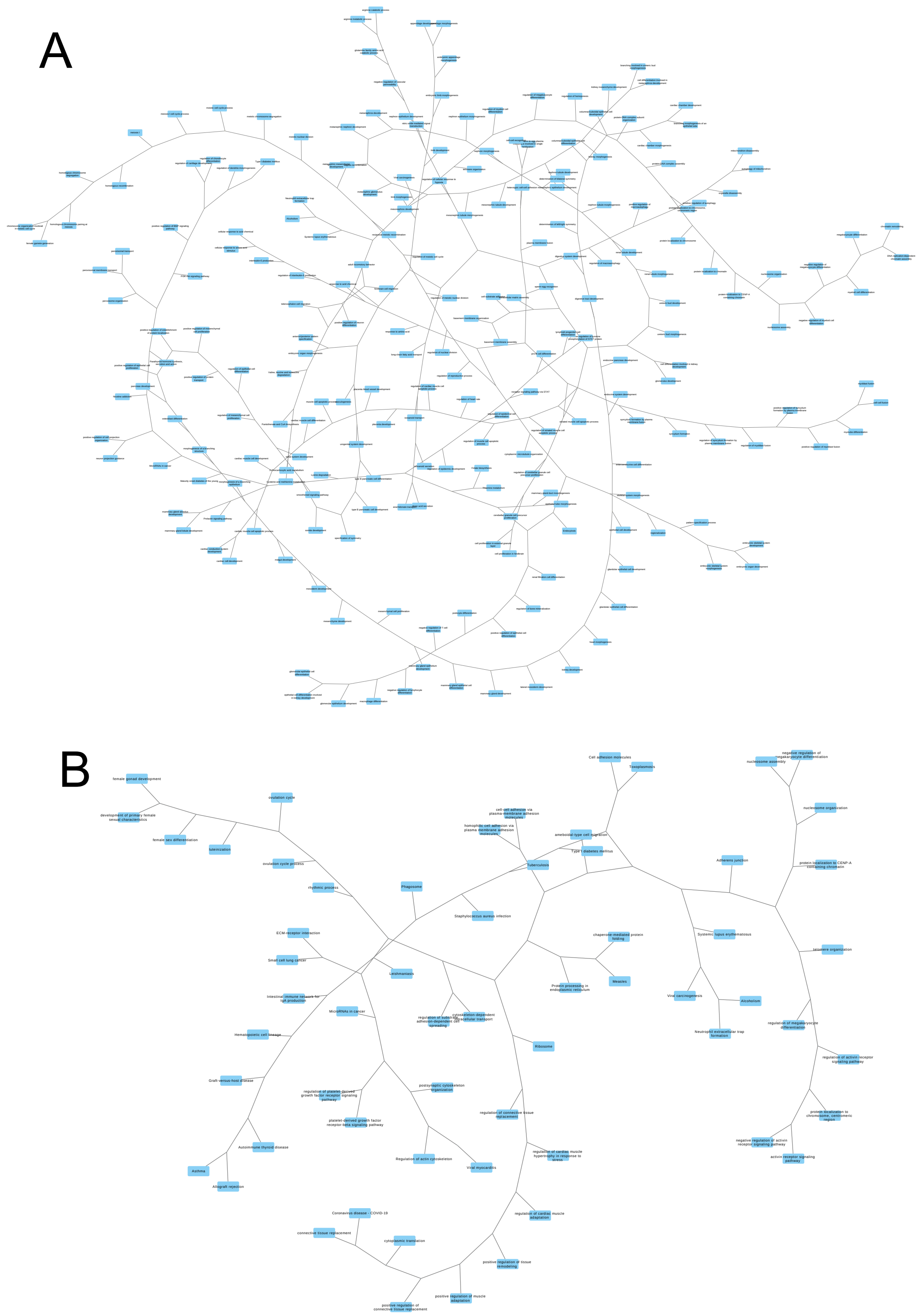

Figure S3. Network representation of selected functions aimed at constructing potential regulatory models. We identified features that exhibited covariation with the elements responsible for functional enrichment. A) LUAD. B) LUSC.
